# From Sodium Storage Mechanism to Design of High-Capacity Carbon-Based Anode: A Review

**DOI:** 10.3390/ma18102248

**Published:** 2025-05-13

**Authors:** Yujun Zhou, Zhongrong Shen

**Affiliations:** 1 Xiamen Tungsten Co., Ltd., 22F, Building A, Tefang Center, No. 81 Zhanhong Road, Siming District, Xiamen 361009, China; 2 Xiamen Key Laboratory of Rare Earth Photoelectric Functional Materials, Xiamen Institute of Rare Earth Materials, Haixi Institutes, Chinese Academy of Sciences, Xiamen 361021, China; z-shen@fjirsm.ac.cn

**Keywords:** sodium-ion batteries, carbon-based anode, hard carbon, sodium storage mechanism, microstructure design

## Abstract

Sodium-ion batteries (SIBs) have emerged as a viable alternative to lithium-ion technologies, with carbon-based anodes playing a pivotal role in addressing key challenges of sodium storage. This review systematically examines hard carbon as the premier anode material, elucidating its dual sodium storage mechanisms: (1) sloping capacity (2.0–0.1 V vs. Na^+^/Na) from surface/defect adsorption and (2) plateau capacity (<0.1 V) via closed-pore filling and pseudo-graphitic intercalation. Through critical analysis of recent advancements, we establish that optimized hard carbon architectures delivering 300–400 mAh/g capacity require precise coordination of pseudo-graphitic domains (d_002_ = 0.36–0.40 nm) and <1 nm closed pores. This review ultimately provides a design blueprint for next-generation carbon anodes, proposing three research frontiers: (1) machine learning-guided microstructure optimization, (2) dynamic sodiation/desodiation control in sub nm pores, and (3) scalable manufacturing of heteroatom-doped architectures with engineered pseudo-graphitic domains. These advancements position hard carbon anodes as critical enablers for high-performance, cost-effective SIBs in grid-scale energy storage applications.

## 1. Introduction

Sodium-ion batteries (SIBs), as a potential alternative or complementary technology to lithium-ion batteries (LIBs), offer advantages including low cost, resource abundance, and independence from scarce metal resources. They also demonstrate exceptional high-rate performance and low-temperature operational capabilities [1], rendering them promising for applications in large-scale energy storage systems, low-speed electric vehicles and two-wheelers, backup power supplies and base stations, as well as hybrid and entry-level electric vehicles. Nevertheless, compared to mature LIB technologies, SIBs still exhibit limitations in capacity retention and cycle stability. The design and development of high-capacity anode materials, as one of the critical components in SIBs, represent a pivotal approach to address the current challenge of low energy density of SIBs [2,3,4]. Although numerous non-carbon-based anode materials [5,6,7,8,9,10,11,12,13,14] have been extensively investigated, this review will focus specifically on carbon-based anodes, given the industrial demand for more stable cycling performance and cost-effective manufacturing in practical applications.

**Figure 1:**
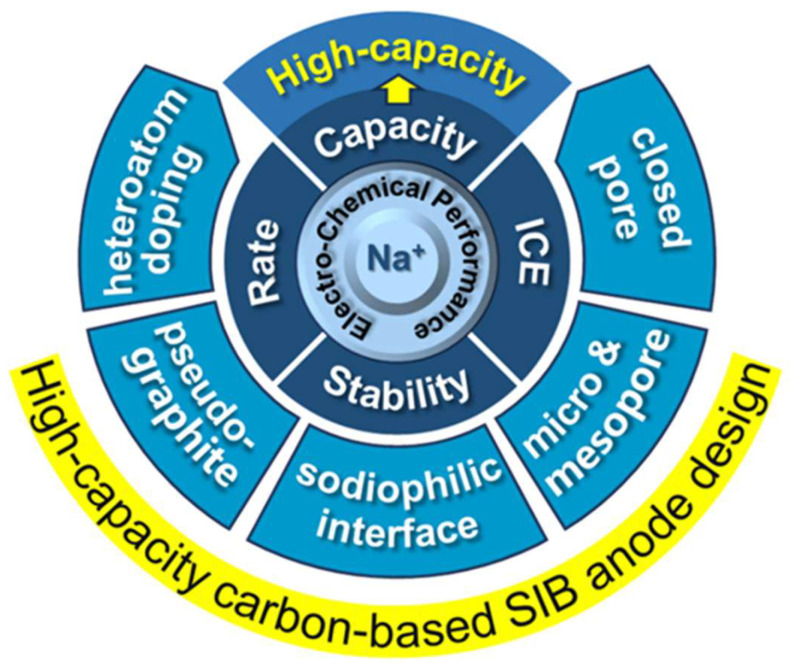
Multiscale design strategies for high-capacity carbon anodes.

**Figure 2:**
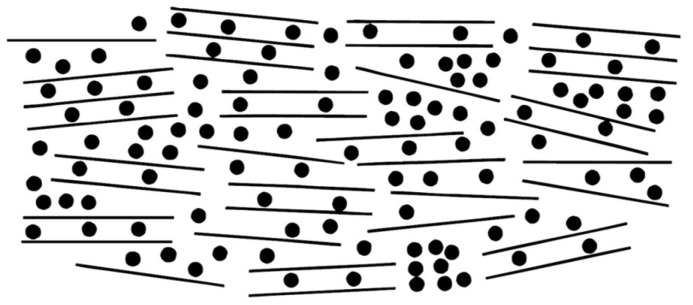
Illustration of sodium ions (black dots) in “house of card” model [24]. Copyright 2000, IOP Publishing.

For comprehensive discussions on non-carbon anode systems, readers are referred to specialized reviews [15,16]. Carbon-based anodes have recently emerged as a research hotspot in SIB development. While numerous reviews on anode materials exist to date, most primarily cater to experienced researchers through detailed methodological descriptions and corresponding electrochemical performance analyses [17,18,19,20,21,22,23]. The fundamental objective of this review diverges by specifically addressing the needs of researchers and graduate students newly entering the field of carbon-based anodes. We have deliberately dispensed with conventional formalities and superfluous contextual preliminaries characteristic of traditional reviews, instead presenting a concise yet comprehensive analysis. To facilitate a systematic understanding for researchers entering this field, this review will conduct the following:

(1) Elucidate sodium storage mechanisms in carbon matrices, with emphasis on how microstructural and compositional characteristics (e.g., closed pores, graphitic domains, mesopores/micropores, heteroatom doping, and sodiophilic interfaces) govern storage behavior (Figure 1);
(2) Establish structure–performance relationships between carbon microarchitecture and sodium storage capacity;
(3) Analyze recent advances in synthesis methodologies for precise microstructure control and strategies for capacity enhancement;
(4) Discuss appropriate characterization techniques for different carbon architectures while addressing common analytical pitfalls;
(5) Provide perspectives on future development directions for high-capacity carbon anodes, proposing actionable pathways for advancing SIB technology.

Through this structured approach, we aim to equip readers with a clarified roadmap for future research directions in carbon-based anode development for sodium-ion battery systems.

## 2. Mechanism for Sodium-Ion Storage

The most prevalent storage mechanism in reversible sodium-ion batteries operates through the rocking-chair principle, where sodium ions serve as charge carriers migrating between cathode and anode during redox reactions. This ion shuttle drives electron flow in the external circuit, enabling interconversion between chemical and electrical energy. Optimal systems should maintain sodium ions within host structures—materials requiring both sufficient sodium accommodation capacity and efficient ion transport pathways. Stevens and Dahn addressed this dual requirement through their proposed “house of cards” structural model (Figure 2) [24]. In this architecture, carbon atomic “bricks” form the walls and corridors of the card-house structure. Current research focuses on engineering these carbon building blocks into optimized configurations that simultaneously maximize sodium storage density and provide unobstructed sodium-ion transport pathways.

Natural precursors such as biomass [25,26,27,28,29] and anthracite [30,31,32,33,34] can spontaneously form sodium-accommodating “host structures” through high-temperature carbonization. However, these naturally derived precursors inherently lack precise control over their resultant carbon microarchitectures. While optimization strategies like pre-oxidation treatments [35,36], controlled carbonization protocols [37,38], and carbonization atmosphere regulation [39] have proven effective in enhancing electrochemical performance, the fundamental relationship between carbon microstructure and sodium storage mechanisms remains a subject of ongoing investigation and debate. The inherent compositional and structural variabilities of natural precursors during pyrolysis often lead to performance degradation through two primary pathways: (i) irreversible sodium trapping at structural defects, which reduces initial Coulombic efficiency (ICE) and diminishes full-cell capacity. This has driven widespread adoption of pre-sodiation strategies in both research and industrial applications to mitigate ICE limitations [40,41,42,43]. (ii) Partial graphitization-induced contraction of interlayer spacing, which compromises ionic conductivity and simultaneously degrades both capacity and rate capability. These challenges highlight the promise of rationally designed carbon composite architectures for high-capacity applications, though precise molecular-level structural engineering remains technically demanding. Recent advances focus on constructing closed-pore and graphitic domain models through precursor composition design and microstructure manipulation, aiming to establish explicit structure–performance correlations [44,45,46,47]. While these pioneering studies provide novel insights into carbon anode optimization, the realization of ideal sodium storage configurations remains elusive. This review systematically analyzes current research efforts across various model systems to elucidate critical structure–energy storage relationships.

### 2.1. Graphite

Graphite serves as the foundational carbon material in energy storage systems, with its natural interlayer spacing (~0.335 nm) being optimally suited for smaller Li^+^ ions (ionic radius: 0.076 nm). This configuration enables a theoretical lithium storage capacity of 372 mAh/g, typically represented by the simplified formula LiC_6_. However, larger alkali ions such as Na^+^ (0.102 nm) and K^+^ (0.138 nm) face significant challenges in overcoming the π–π interactions between graphene layers [48]. While steric hindrance fundamentally limits intercalation of these larger ions, their insertion behavior is ultimately governed by the thermodynamic–kinetic interplay involving the following [49,50,51,52,53,54]: (i) diffusion energy barriers; (ii) formation of stable intercalation phases; and (iii) solvation shell effects. For sodium ions specifically, intercalation into graphite induces severe structural degradation due to lattice mismatch, resulting in negligible practical capacity (<35 mAh/g). Interestingly, despite potassium ion’s larger ionic radius and more pronounced volumetric expansion (~60% vs. 10% for Li^+^), potassium demonstrates superior storage capability (279 mAh/g, represented as KC_8_) through three key mechanisms: (i) lower diffusion barriers enabling faster kinetics; (ii) dynamic interlayer spacing accommodation during insertion; and (iii) reversible phase transitions maintaining structural integrity. Additionally, solvation shell effects play a critical role: potassium ions, due to their lower solvation energy, more readily shed their solvation shells during intercalation, reducing their effective ionic diameter. This mitigates intercalation-induced stress. In contrast, sodium ions’ stronger solvation interactions often lead to co-intercalation of solvent molecules into graphite layers, exacerbating structural expansion and irreversible phase transformations [55].

**Figure 3:**
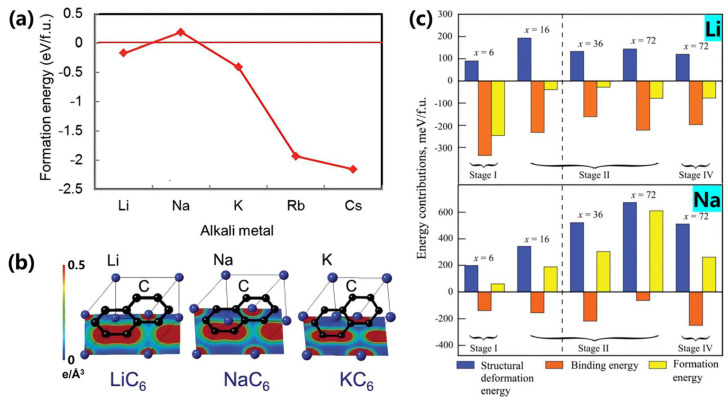
(**a**) Calculated formation energies of AMC_6_ (where AM is alkali metal) [56]; Copyright 2017, Royal Society of Chemistry. (**b**) The greater electron density of LiC6 indicating the covalent bonding contribution between C and Li [56]; Copyright 2017, Royal Society of Chemistry. (**c**) Contributions to the formation energy of LiCx and NaCx in different stages of GICs [57]; Copyright 2019, Royal Society of Chemistry.

Moriwake et al. [56] employed density functional theory (DFT) calculations incorporating van der Waals functionals to investigate the formation energy of AmC_6_ configurations (where Am = alkali metal) in graphite intercalation compounds (GICs). Figure 3 reveals that alkali metal–carbon formation energies become progressively more stable (negative) from Na to Cs, driven by ionic radius expansion and strengthened ionic bonding interactions. Specifically, sodium ions (Na^+^) demonstrate positive formation energy values, indicating thermodynamic instability of Na–GICs. In contrast, larger alkali metals (K, Rb, Cs) exhibit progressively negative formation energies (Figure 3a). Notably, lithium deviates from this trend due to its smaller ionic radius and distinct Li–C covalent interactions (Figure 3b), making sodium the most challenging alkali metal for graphite intercalation.

Mollenhauer et al. [57] further analyzed formation energy differences in Li/Na/K–GICs through first-principle calculations (Figure 3c), defining total formation energy as the sum of structural deformation energy and metal–carbon binding energy. The strong covalent interaction between small Li^+^ ions and carbon atoms creates significant orbital overlap, while larger Na atoms exhibit negligible covalent bonding. This fundamental distinction explains why lithium breaks the alkali metal size trend in GICs.

**Figure 4:**
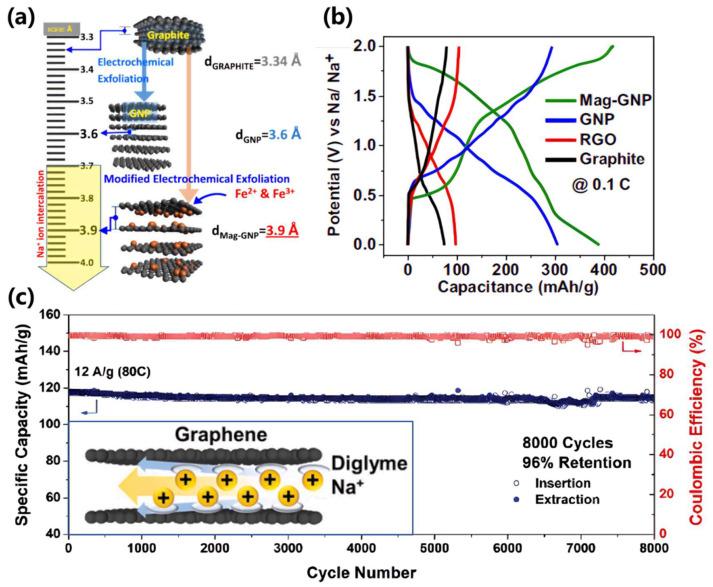
(**a**) Modified electrochemical exfoliation to generate graphene nanoplatelet (GNP) and magnetite nanoparticle-coated GNP (Mag-GNP) and with interlayer distances of 3.6 Å and 3.9 Å, respectively [58]; Copyright 2024, Wiley-VCH GmbH. (**b**) The galvanostatic charge–discharge curves for Mag-GNP, GNP, RGO, and graphite in half-cells [58]; Copyright 2024, Wiley-VCH GmbH. (**c**) Diglyme-assisted sodium-ion intercalation for a high-capacity retention after 8000 cycles, where inset demonstrates that a diglyme solvent shell encapsulating a sodium ion acts as a “nonstick” coating to realize the ultrafast Na^+^ ion insertion graphite layers [59]; Copyright 2016, American Chemical Society.

The inherent instability of Na–GICs—stemming from weak binding energy and substantial structural deformation requirements—precludes direct application in sodium-ion battery anodes requiring high-rate cycling stability, reversible intercalation/deintercalation kinetics, or structural integrity maintenance over extended cycles. However, interlayer spacing expansion strategies can effectively reduce deformation energy and enhance capacity to 200–300 mAh/g. Current approaches include the following: (i) intercalation-mediated expansion: inserting metal ions (Mag-GNP, as shown in Figure 4a) [58], solvent molecules, or polymers to enlarge interlayer spacing while enhancing sodium binding energy; and (ii) chemical oxidation: treating graphite with strong oxidizers (HNO_3_, H_2_SO_4_) to generate oxygen-containing functional groups (carboxyl, epoxy), expanding interlayer spacing to 0.4–0.45 nm. Reduced graphene oxide (rGO; labeled as Graphene in Figure 4c) achieves reversible capacities of ~100 mAh/g under ultrahigh current densities (30 A/g, equivalent to ~80C rate), while maintaining 98% capacity retention over 8000 cycles [59]. These methodologies will be further discussed in subsequent sections addressing heteroatom doping and pseudo-graphitic domain engineering.

### 2.2. Pseudo-Graphite

Compared to graphite anodes discussed in the preceding section, hard carbon is recognized as the optimal anode material for sodium-ion batteries (SIBs), delivering a specific capacity of 300–400 mAh/g [60]. Concurrently, achieving lower sodium insertion voltage plateaus has become a critical research focus to enhance full-cell operating voltage. The observed plateau capacity is predominantly attributed to contributions from pseudo-graphitic domains and closed pores. However, the stochastic formation of these structures during precursor carbonization makes it inherently challenging to definitively distinguish their respective sodium storage mechanisms. Recent efforts employ molecular design strategies to synthesize hard carbons with enriched pseudo-graphitic domains or closed-pore architectures, aiming to elucidate their distinct electrochemical signatures [61].

Pseudo-graphitic domains are characterized by interlayer spacings (d_002_) of 0.36–0.40 nm, facilitating sodium-ion intercalation/deintercalation and manifesting as voltage plateaus [62]. Notably, interlayer spacing alone cannot serve as a definitive criterion for pseudo-graphitic identification. Karunarathna et al. demonstrated this limitation through Fe^2+^/Fe^3+^-intercalated graphite via electrochemical exfoliation, achieving expanded interlayer spacings (0.36–0.39 nm) without exhibiting the characteristic low-voltage plateau (<0.1 V vs. Na^+^/Na) observed in hard carbons (Figure 4b). This underscores that pseudo-graphitic structures in hard carbons derive not merely from increased d_002_ spacing but also from structural distortions induced by heteroatom doping (N, S, O, P, etc.) [63], irregular carbon layer stacking [55], or lattice defects [17]. These structural modifications enhance electrochemical performance by (i) providing additional active sites for sodium storage [64,65], (ii) improving structural resilience during cycling [1], and (iii) optimizing sodium diffusion pathways [66]. In contrast to closed-pore storage mechanisms, pseudo-graphitic domains primarily operate through intercalation-based sodium storage. This process involves reversible insertion/extraction of sodium ions between carbon layers, mediated by dynamic interactions between Na^+^ and the carbon matrix. The sodium storage capacity and kinetics of pseudo-graphitic structures are critically dependent on (i) interlayer spacing optimization, (ii) structural defect engineering, and (iii) electron/ion transport synergy. Well-designed pseudo-graphitic architectures enable rapid sodium-ion diffusion while maintaining structural integrity, significantly enhancing rate capability and cycling stability compared to conventional graphite-based systems.

**Figure 5:**
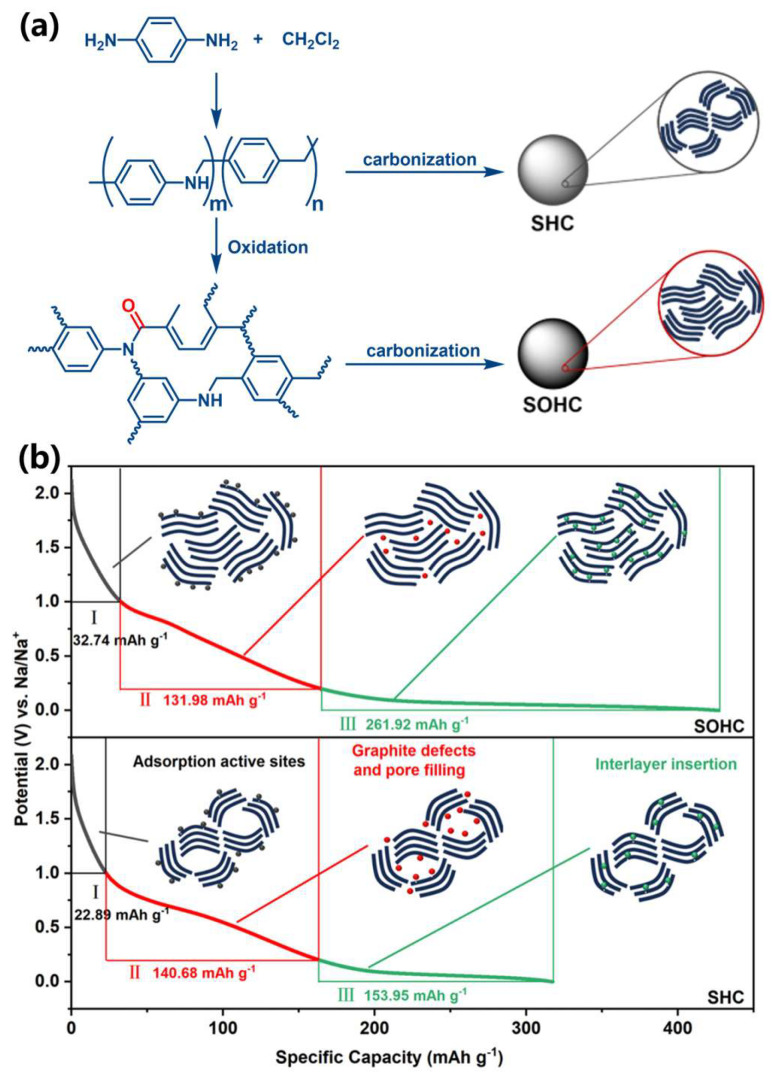
(**a**) Schematic illustration depicting the synthesis of hard carbon microspheres with architecturally designed graphite-rich domains using polymer precursors [69]; Copyright 2024, Elsevier. (**b**) Comparative analysis illustrating the enhanced low-voltage plateau capacity contribution from graphitic domains in hard carbon anodes during sodium-ion storage processes, benchmarked against conventional closed-pore carbon materials in half-cell configurations [69]; Copyright 2024, Elsevier.

The sodium-ion intercalation behavior within pseudo-graphitic layers involves diffusion-limited kinetics, typically manifested in low-voltage plateau regions where diffusion coefficients exhibit rapid decline [67]. This phenomenon arises from the constrained ion transport through narrow interlayer spacings during NaCx compound formation, resulting in distinct voltage plateaus below 0.1 V [68]. In recent mechanistic studies, Zhao et al. [69] developed a two-stage synthesis protocol for hard carbon microspheres: First, halogenated amination of p-phenylenediamine/dichloromethane co-precursors generates polymeric intermediates, which subsequently undergo differentiated carbonization—either oxidative polymerization-assisted (SOHC) or conventional pyrolysis (SHC)—as depicted in Figure 5a. The pseudo-graphitic-rich microspheres delivered a total capacity of 339 mAh/g, with 262 mAh/g originating from the plateau region associated with sodium storage in pseudo-graphitic domains (Figure 5b), conclusively demonstrating their dominant role in plateau capacity. Notably, these pseudo-graphitic domains differ fundamentally from graphite: (i) Their expanded interlayer spacing (~3.5–4.0 Å vs. ~3.35 Å in graphite) mitigates strain during Na+ intercalation, and (ii) defects/curvature and interconnected nanopores provide low-energy adsorption sites and ion pathways, enabling synergistic intercalation–surface adsorption. In contrast, graphite’s defect-free stacking and narrow interlayer spacing thermodynamically disfavor stable Na–GIC formation, as evidenced by its positive formation energy. This structural disorder-to-order dichotomy directly explains the capacity disparity.

Wang et al. [70] further advanced this understanding through pre-oxidation treatment of lignin derivatives. The introduced oxygen-containing functional groups effectively truncated long side chains in lignin’s aromatic units, promoting crosslinking during pyrolysis to form three-dimensional network structures. This approach enabled precise control over pseudo-graphitic/closed-pore ratios, revealing that increased pseudo-graphitic content significantly enhances both plateau capacity and rate performance. The expanded interlayer spacing in pseudo-graphitic structures (compared to conventional graphite) reduces structural deformation energy requirements during sodium intercalation, fundamentally altering sodium diffusion kinetics and storage behavior.

To optimize sodium storage capacity and kinetics, three primary pseudo-graphitic engineering strategies have emerged: (i) thermal treatment optimization: precise control of carbonization temperatures (800–1600 °C) and heating rates (2–10 °C/min) enables tailored graphitization degrees and pseudo-graphitic domain formation [37,71]; (ii) heteroatom doping: strategic incorporation of metal ions (e.g., Fe, Co) or non-metallic elements (e.g., N, S) enhances graphitization efficiency and stabilizes pseudo-graphitic architectures [4,72]; and (iii) pre-oxidation engineering: controlled oxidation of precursors using O_2_ or H_2_O_2_ introduces oxygen functional groups that modify pyrolysis pathways, promoting crosslinking reactions that simultaneously increase pseudo-graphitic domains and functional closed pores [73,74]. These synergistic approaches enable coordinated optimization of pseudo-graphitic domains and closed-pore architectures, achieving enhanced sodium storage capacity (300–400 mAh/g) with improved rate capability (>80% capacity retention at 2C).

### 2.3. Closed Pores

**Figure 6:**
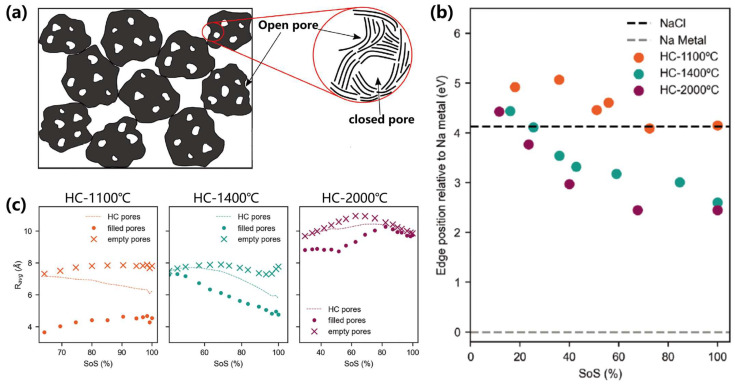
(**a**) Illustration of the cross section of hard carbon particles (left) and possible microstructures for closed pores and open pores; and (**b**) sodiation-dependent Na K-edge shifts for three HC variants, with dashed reference lines indicating metallic Na (gray) and NaCl (black); (**c**) The average pore sizes of the HC pores, filled pores, and empty pores as a function of state of sodiation (SoS) based on the plateau region for hard carbon prepared from 1100 °C to 2000 °C [75]; Copyright 2023, Wiley-VCH GmbH.

In describing the microscopic molecular or elemental composition of hard carbon materials, researchers typically seek definitive theoretical models for mechanistic understanding. A conventional conceptualization portrays closed/open pores in hard carbon as idealized void spaces (Figure 6a) [75], where closed pores provide sodium storage “containers” bounded by carbon walls. However, the actual structural complexity far exceeds these simplified representations, and the precise definition of closed pores remains debated. Key unresolved questions include: (i) Are closed pores formed by wrinkled graphene stacking? (ii) Do they originate from localized carbon defects? (iii) Could folded graphite sheets or fullerene-like cavities (C60, C70) qualify as closed pores? Even with comparable pore sizes detected via small-angle X-ray scattering (SAXS), microstructural variations inevitably lead to divergent sodium storage behaviors. Nevertheless, consensus has emerged that closed pores are predominantly filled during low-voltage plateau regions. Current research focuses on engineering optimized closed-pore architectures by controlling pore size distribution, carbon wall crystallinity, defect density, and sodium-filling mechanisms.

Building on the structural complexity of hard carbon pores, sodium storage mechanisms in the sloping (≈0.1–2.5 V) and plateau (≈0–0.1 V) regions are critically influenced by pore architecture and defect dynamics. During the sloping region, sodium ions predominantly adsorb at defect-rich sites (e.g., vacancies, edges) in smaller pores, retaining an ionic state as evidenced by minimal XANES edge shifts in low-temperature HCs (e.g., HC-1100 °C). In contrast, the plateau region involves non-uniform filling of larger pores in high-temperature HCs (e.g., HC-2000 °C), where reduced defect density facilitates the formation of pseudo-metallic sodium clusters, reflected in XANES edge positions approaching metallic sodium [76]. Concurrently, sodium intercalation between graphene layers contributes to plateau capacity, particularly in HCs with expanded interlayer spacing (e.g., HC-1100 °C, d_002_ ≈ 3.78 Å). These findings highlight how pore size distribution and defect engineering dictate sodium speciation (ionic vs. metallic-like), guiding the design of optimized hard carbon anodes for enhanced sodium storage efficiency.

Recent breakthroughs demonstrate these principles. Song et al. [77] ingeniously employed zinc gluconate (ZG) precursors with ZnO templates to construct 0.45–4 nm open frameworks, subsequently converted to closed pores through high-temperature carbonization (Figure 6b). Sodium clusters formed within these closed pores during low-voltage plateaus (<0.1 V), achieving a remarkable capacity of 481.5 mAh/g with 81% plateau contribution (389 mAh/g). This performance enhancement correlated with increased closed-pore density at elevated carbonization temperatures. Complementary studies by Tang et al. revealed critical size effects [25,78]. Controlled annealing (1100–2000 °C) progressively enlarged closed pores from 5.1 Å to 9.2 Å while enhancing sodium storage capacity. Optimal performance occurred at <1 nm pore sizes, where reduced defect density and minimized sodium surface exposure synergistically lowered deposition overpotentials and improved rate capability. While DFT simulations [79] identified maximal sodium adsorption energy at 0.45 nm pores, experimental data indicate larger pores (5.1–9.2 Å) and improve practical capacity through two synergistic mechanisms: (1) accommodating Na^+^ clusters despite reduced per-ion adsorption energy; and (2) balancing pore density and ion diffusion kinetics. Notably, sodium adsorption energy progressively decreases with increasing closed-pore size, particularly beyond critical thresholds. For instance, 0.72 nm pores retain a moderate adsorption energy of −3.07 eV [79], but this value plummets to −0.13 eV at 0.9 nm, indicating severe efficiency loss in sodium storage capacity at larger dimensions. This highlights the necessity of designing hierarchical pore systems where sub-nanometer pores (0.45 nm) maximize adsorption strength, while slightly larger pores (5.1–9.2 Å) enhance structural stability and ion transport.

**Figure 7:**
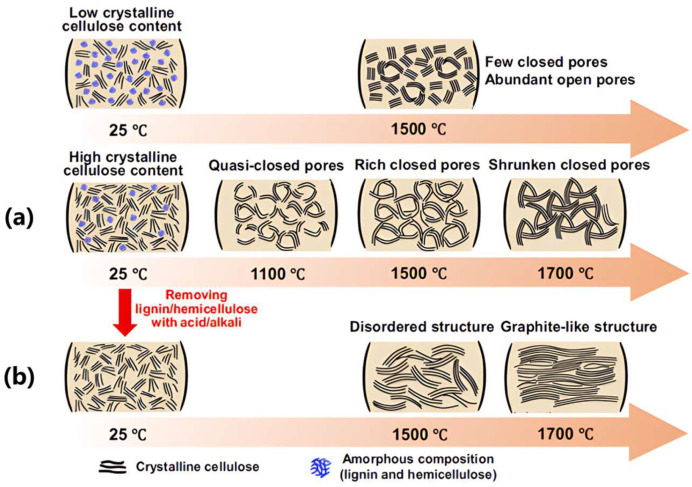
Microstructure design of closed pores and pseudo-graphite by (**a**) annealing temperature or (**b**) precursors [25]; Copyright 2023, Spring Nature.

Beyond capacity enhancement, closed-pore architectures mitigate volume expansion during cycling through mechanical buffering. Effective closed-pore formation requires sufficient graphitic domain lengths and curvature-capable carbon structures. Figure 7 illustrates this principle through cellulose carbonization [25]: (i) amorphous lignin components prevent excessive graphitization while creating active sites; (ii) crystalline cellulose domains form elongated graphitic walls enclosing closed pores (Figure 7a); and (iii) removal of lignin disrupts pore formation, yielding non-porous carbon (Figure 7b). These findings validate the dual-control strategy of precursor composition (cellulose/lignin ratio) and annealing temperature (800–1600 °C) for closed-pore engineering [4,44,80,81].

### 2.4. Micro- and Mesoporous Structures

Beyond the dominant plateau capacity, carbon anode materials exhibit additional sloping capacity contributions within the 2.0–0.1 V vs. Na^+^/Na voltage range. While some researchers propose an “intercalation-adsorption” mechanism attributing sloping capacity to Na^+^ intercalation in graphitic-like layers and plateau capacity to micropore filling/deposition [24,82], the prevailing consensus associates sloping capacity with Na^+^ adsorption at defect sites and carbon surfaces (including micro-/mesoporous interfaces), supported by substantial theoretical and experimental evidence [19,83]. This adsorption-dominated storage mechanism establishes a fundamental trade-off: While higher defect density and surface area enhance sloping capacity through increased adsorption sites, they simultaneously introduce more irreversible sodium trapping at structural defects and metastable interfaces, leading to significant initial Coulombic efficiency (ICE) deterioration.

The strategic design of mesoporous architectures enhances electrochemical performance through improved ion transport kinetics. Yang and Lv et al. [84] demonstrated that optimized mesoporosity in hard carbons increases active site accessibility and sodium diffusion rates. However, micro-/mesoporous structures inherently reduce tap density and introduce surface defects that irreversibly trap sodium ions, thereby compromising ICE. Their study revealed a strong negative correlation between sloping capacity contribution (>30%) and ICE values (<80%), highlighting the critical need for defect density modulation in pore engineering. This creates a critical design challenge: balancing pore architecture (size/distribution) with defect engineering to maximize capacity while minimizing irreversible losses. Recent studies suggest that the sloping region’s quasi-metallic sodium clusters exhibit partial reversibility, with ICE losses primarily originating from (i) permanent sodium entrapment at deep defect sites and (ii) SEI formation on newly exposed carbon surfaces during pore filling.

Pore entrance diameter significantly impacts sodium transport dynamics. While narrow micropore entrances (<0.7 nm) may hinder solvated Na^+^ ingress, desolvated ions can permeate through ultramicropores (<0.5 nm) via size-exclusion effects, enabling high-rate performance. Advanced pore engineering strategies include the following: (i) chemical vapor deposition (CVD) for pore filling in activated carbons; (ii) polyethylene glycol (PEG)-assisted mesopore sealing; and (iii) pitch-derived carbon coatings for selective pore modification [66,85,86]. These approaches effectively suppress detrimental open porosity while preserving functional micropores.

Hu et al. [78] provided mechanistic clarity through combined DFT calculations and experimental validation. DFT simulations of alkali metal edge adsorption on graphene revealed a zigzag Na configuration with gradually decreasing adsorption energy, consistent with sloping region behavior. Bader charge analysis of sodiated wedge pores showed ≈20% valence electron density transfer to the carbon matrix, localized primarily within pores. This electron redistribution, coupled with density of states (DOS) profiles, confirms the quasi-metallic nature of confined sodium species. The sloping region’s storage mechanism involves rapid Na^+^ intercalation and surface diffusion processes. The steep voltage decline reflects efficient sodium insertion into hard carbon’s nanostructured pores, forming metastable sodiation phases. This process is governed by nanoscale porosity facilitating fast ion transport, optimized particle size distributions reducing diffusion paths, and defect engineering enhancing adsorption kinetics. During discharge, sodium ions dynamically migrate and accumulate within interconnected pore networks, forming stable interfacial layers that enable high-capacity retention even at elevated rates (>2C). The synergistic combination of rapid ion mobility and confined metallic sodium clusters underpins the sloping region’s exceptional rate capability and cycling stability.

The catalytic role of carbon surface defects in electrolyte decomposition and subsequent formation of carbonaceous species during electrochemical cycling remains a critical concern. In sodium-ion batteries employing hard carbon anodes, carbonate-based electrolytes undergo reductive decomposition, forming a heterogeneous solid electrolyte interphase (SEI) composed of inorganic compounds (e.g., Na_2_CO_3_, NaO_2_) and organic derivatives (e.g., alkyl/polycarbonates) [87]. The porous architecture and high surface area of hard carbons exacerbate localized electrolyte concentration gradients, promoting deep reduction reactions that deposit carbonaceous byproducts at defect sites and pore interfaces. This process exhibits strong dependence on electrolyte composition, cycling conditions (rate/temperature), and electrode surface states.

Analogous to lithium metal batteries where inhomogeneous Li deposition induces dendrite formation and short-circuit risks [88], sodium-ion systems face similar challenges during hard carbon sodiation. Carbonaceous species accumulation modifies interfacial properties through dual mechanisms: increased surface heterogeneity elevates interfacial impedance and promotes dendritic sodium growth, while mechanical stress from dendrite-embedded structures triggers SEI fracture. These conductive byproducts further accelerate dendrite propagation through enhanced electron transport pathways, significantly raising internal short-circuit probabilities.

Mitigation strategies focus on interrupting the carbonaceous deposition cycle through coordinated approaches. Electrolyte engineering via fluorinated solvents or high-concentration salts redirects decomposition pathways, while artificial SEI layers (e.g., polymer-inorganic composites) suppress parasitic reduction reactions at defect sites [89]. Such synergistic modifications demonstrate enhanced interfacial stability without compromising electrochemical performance, providing a viable route toward safer sodium-ion battery operation.

## 3. Synthesis and Characterization

Accurate characterization of the microstructure of hard carbon is critical for elucidating sodium-ion storage mechanisms and guiding the design of high-performance hard carbon materials. To systematically compare the capabilities of different techniques, Table 1 summarizes conventional characterization methods categorized by structural and electrochemical analyses, along with their physical significance. These methods provide complementary insights into the crystallinity, porosity, surface chemistry, and dynamic behavior of hard carbon during sodiation/desodiation.

Current characterization techniques for hard carbon microstructures are further classified into three operational modes: ex situ, in situ, and operando, each addressing distinct scientific questions.

Ex situ characterization involves analyzing post-cycled hard carbon electrodes extracted from sodium-ion batteries to infer structural changes under different electrochemical states. For instance, ex situ X-ray diffraction (XRD) is employed to study sodium-ion intercalation behavior in graphitic domains, though significant shifts in the characteristic (002) peak positions are rarely observed during most charge–discharge stages [26,90,91]. Ex situ Raman spectroscopy, which monitors changes in the intensity ratio of the defect-induced D-band to the graphitic G-band (I_D_/I_G_), offers higher sensitivity for tracking disorder evolution during sodiation/desodiation [26,45,69]. However, these ex situ methods require post-testing sample cleaning, which risks damaging the solid electrolyte interface (SEI) layer and fails to capture dynamic structural responses under real-world operating conditions [92].

In situ characterization enables real-time monitoring of microstructural evolution under simulated battery environments. Techniques such as in situ XRD track characteristic peak shifts to resolve sodium-ion storage pathways (e.g., intercalation in graphitic domains or adsorption at defect sites) [71,93]. In situ Raman spectroscopy reveals dynamic changes in D-/G-band intensities, correlating with sodium-ion adsorption at defects or interlayer spaces [94,95]. In situ transmission electron microscopy (TEM) directly visualizes sodium deposition and cluster formation within closed pores [96,97], while in situ ^23^Na nuclear magnetic resonance (NMR) probes the formation of quasi-metallic sodium in closed pores via Knight shift analysis [78,98]. Despite their proximity to realistic electrochemical conditions, challenges remain in replicating critical parameters such as localized pressure and temperature gradients.

Operando characterization advances this by integrating real-time structural analysis with full-cell operation, providing the most authentic data on dynamic structural–electrochemical interplay. Representative methods include operando Raman spectroscopy [84,99], operando XRD [61,100], and operando NMR [101]. These techniques synchronize electrochemical signals (e.g., voltage/current) with microstructural evolution, establishing quantitative structure–kinetics–capacity relationships to guide material optimization.

## 4. Conclusions and Perspectives

Recent advancements in carbon-based anode materials have demonstrated remarkable progress through diversified design strategies, as systematically outlined in Table 2. Cutting-edge innovations now enable researchers to achieve precise regulation of critical parameters—including precursor selection, pretreatment conditions, and carbonization protocols—to tailor carbon architectures for optimized sodium storage performance.

**Figure 8:**
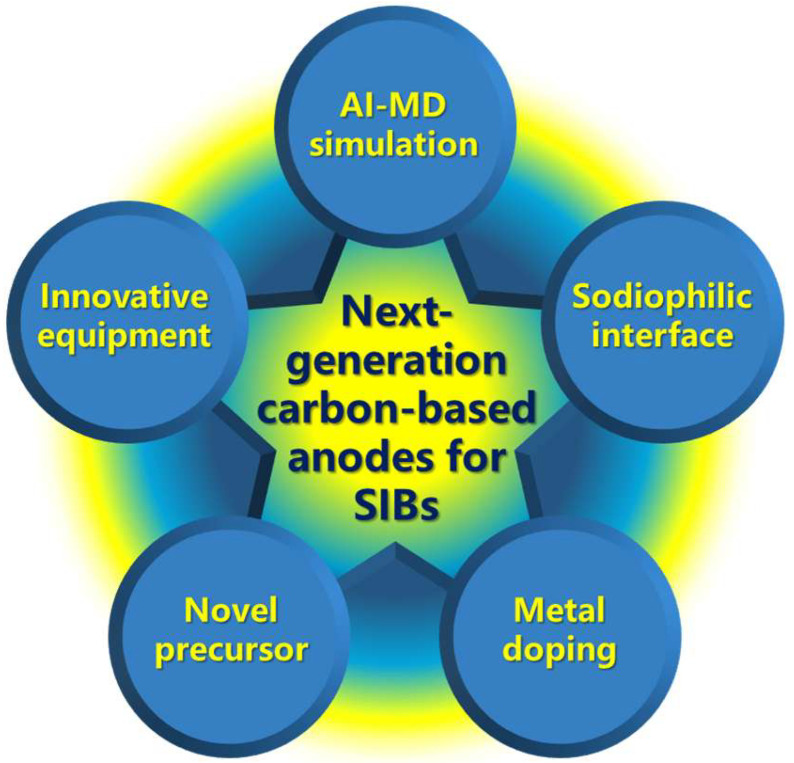
Perspectives for future research in next-generation carbon-based anodes.

Current breakthroughs underscore that the integration of closed-pore architecture design with pseudo-graphitic domain engineering forms the foundation for high-performance carbon anodes, effectively balancing capacity enhancement and rate capability optimization. The strategic development of closed-pore-enriched hard carbons, which synergistically combine high reversible capacity (>480 mAh/g) with exceptional cycling stability, has established itself as the prevailing paradigm for next-generation sodium-ion batteries. Looking forward, priority optimization targets for hard carbon anodes include the following: (i) improving initial Coulombic efficiency (>90%), (ii) achieving sub-nanometer precision in closed-pore sizing, (iii) implementing defect engineering to mitigate irreversible sodium trapping, (iv) reducing sodium deposition overpotential (<10 mV), and (v) minimizing non-functional surface areas. Building on existing advancements in precursor engineering and thermal processing, emerging methodologies—particularly machine learning-guided microstructure optimization and sodiophilic interface design—show strong potential to propel carbon anode technology toward practical applications (see Figure 8).

(a) AI-accelerated Molecular Dynamics (AI-MD) for sodium storage mechanism

Current mechanistic studies on sodium storage in hard carbon anodes primarily rely on conventional computational approaches, including density functional theory (DFT), Monte Carlo (MC) thermodynamic simulations, and molecular dynamics (MD). While effective for analyzing equilibrium microstructures, these methods face limitations in modeling dynamic sodium transport through complex porous architectures across spatiotemporal scales. Recent breakthroughs in machine-learned interatomic potentials [102], such as Gaussian approximation potentials (GAPs) trained on DFT data [103], offer transformative solutions by maintaining quantum-mechanical accuracy while enabling orders-of-magnitude efficiency gains for large-scale simulations [104]. This computational revolution holds critical significance for establishing a comprehensive theoretical framework of sodium storage mechanisms, particularly in capturing dynamic processes like Na diffusion in disordered carbon frameworks and voltage-dependent intercalation energetics. Future integration of these ML-driven approaches with multiscale modeling and experimental characterization promises accelerated discovery of carbon anode materials with tailored pore architectures and enhanced sodium storage performance.

(b) Innovative equipment with multi-parameter optimization

Pioneering work by Zhang and Wu [105] demonstrates that instantaneous sintering thermal pulse technology enables precise control over local graphitization degree and closed nanopore growth in hard carbons. This approach not only extends low-voltage plateau duration but also enhances energy density. Inspired by graphite’s sp^2^-to-sp^3^ transition under ultrahigh pressure, an advanced hot-pressing apparatus could revolutionize carbon microstructure control. Furthermore, this methodology permits low-temperature fabrication of heteroatom-doped carbons with high carbonization degrees, effectively mitigating dopant evaporation during conventional high-temperature treatments.

(c) Sodiophilic modification for closed pores

As established in previous sections, closed pores < 1 nm facilitate optimal sodium deposition through reversible adsorption mechanisms, while larger pores often degrade capacity due to irreversible Na trapping. To harness the potential of expanded pores (1–2 nm) in hard carbons, strategic precursor modification through metal doping emerges as a promising yet underexplored approach. Recent advances in interfacial engineering, such as Ag nanoparticle-decorated hard carbon microspheres [64] and gradient Ag@CNT frameworks [65], demonstrate that sodiophilic sites significantly reduce nucleation overpotentials (8 mV vs. 23.6 mV on Cu) and regulate Na deposition behavior. These studies highlight the critical role of metal–carbon interfaces in guiding oriented Na crystallization and suppressing “dead sodium” formation. However, existing works predominantly focus on surface modifications or 3D conductive scaffolds, leaving the functionalization of internal meso-/macropores within hard carbons largely unexplored. By integrating sodiophilic nanoparticles (e.g., Ag, Sn) into pore walls via precursor doping or in situ reduction, hierarchical architectures could synergize the advantages of size-restricted micropores (<1 nm) and engineered mesopores (1–2 nm). Such dual-scale designs may enable spatially controlled Na plating within interconnected pores, as evidenced by the enhanced pore-filling efficiency in Ag–HC hybrids (capacity: 550 mAh g^−1^ at 1C). Further innovations could draw inspiration from gradient interfacial layers [65] to create directional sodiophilicity gradients within pores, optimizing Na^+^ flux distribution and mitigating dendrite risks. This paradigm shift toward pore-level interface engineering opens new avenues for high-energy-density Na-ion batteries while maintaining structural robustness.

(d) Novel precursor-engineered fabrication of graphite-like materials with controlled closed-pore architectures

Current hard carbon synthesis predominantly relies on empirical trial-and-error approaches, where precursor selection and post-carbonization characterization remain decoupled. To bridge this critical gap, precursor-engineered fabrication emerges as a promising avenue that warrants systematic exploration. Future investigations should focus on establishing explicit correlations between precursor molecular architectures (e.g., crosslinking density, functional group distribution) and their programmed pore-formation behaviors during carbonization. This requires developing in situ characterization platforms to track dynamic pore evolution while strategically modulating precursor chemistry. Such fundamental understanding could unlock two key capabilities: (1) predictive design of closed-pore topologies through precursor molecular editing, and (2) decoupling pore architecture effects from other variables in sodium storage mechanisms. Implementing this paradigm would shift the field from retrospective analysis to forward-looking material engineering [27,44,45,70].

(e) Metal doping to generate pseudo-graphite

While heteroatom doping has been widely adopted for carbon modification (particularly in porous carbons and catalytic materials) [63], conventional hard carbon synthesis at 1300–1600 °C typically causes dopant evaporation. Innovative doping strategies using silicon or transition metals during high-temperature processing offer new pathways for engineering pseudo-graphitic domains and closed-pore architectures. The retained dopants additionally function as overpotential-reducing sites for sodium deposition.

This review systematically examines the structural determinants of sodium storage mechanisms in carbon anodes, while emphasizing the critical yet frequently overlooked role of electrolyte formulation. Beyond inherent electrode characteristics, electrolyte engineering profoundly impacts storage behavior: Ether-based systems facilitate graphite co-intercalation through ternary compounds (t-GICs) [106], whereas carbonate solvents paired with NaPF_6_/NaClO_4_ optimize hard carbon performance despite inherent material compromises [107,108]. Crucially, electrolyte–electrode synergy governs interfacial stability, with ether-derived Na_2_O/NaF-rich SEI layers demonstrating superior dendrite suppression, underscoring the necessity for concurrent optimization of both components in high-performance SIBs.

## Figures and Tables

**Table 1:** Conventional characterization methods for carbon anodes.

Method	Analysis Focus	Physical Significance
X-ray Diffraction (XRD)	Crystalline Structure	Analyzes long-range ordering, interlayer spacing (d_002_), crystallite size, and graphitization degree.
Raman Spectroscopy	Disorders/Defects	Intensity ratio of D-band (defects) to G-band (graphitic, I_D_/I_G_) reflects disorder and defect density.
Scanning Electron Microscopy (SEM)	Morphology	Observes surface morphology, particle size distribution, and mesoporous structures.
Transmission Electron Microscopy (TEM)	Microstructure	High-resolution imaging of nanopores, carbon layer stacking, and local defects.
BET Surface Area and Pore Analysis	Pore Structure	Measures specific surface area, pore size distribution, and pore volume; correlates with ion transport and storage sites.
X-ray Photoelectron Spectroscopy (XPS)	Surface Chemistry	Identifies surface elemental composition, functional groups (e.g., C-O, C=O), and SEI components.
Fourier Transform Infrared Spectroscopy (FTIR)	Chemical Bonds and Functional Groups	Detects functional groups (e.g., oxygen-containing groups) and chemical bonding types.
Selected Area Electron Diffraction (SAED)	Local Crystalline Structure	Determines local crystallinity (graphitic microdomains) or amorphous regions.
Thermogravimetric Analysis (TGA)	Thermal Stability	Evaluates the precursor carbonization process.
True Density Measurement	Bulk Density and Closed Porosity	Measures skeletal density excluding pores; evaluates closed-pore content for Na/Li storage.
Cyclic Voltammetry (CV)	Reaction Kinetics	Redox peaks reveal Na storage mechanisms; peak separation indicates reversibility.
Galvanostatic Charge–Discharge	Capacity and Cycling Performance	Discharge curve slope correlates with storage mechanisms; cycling stability reflects structural robustness.
Electrochemical Impedance Spectroscopy (EIS)	Interface Resistance	High-frequency semicircle (charge transfer resistance) and low-frequency slope (ion diffusion) reveal kinetic limitations.
Nuclear Magnetic Resonance (NMR)	Ion Storage Mechanism	The ^13^C/^23^Na NMR probes ion environments and diffusion in pores/defects.
Small-Angle X-ray Scattering (SAXS)	Nanoscale Porosity	Quantifies pore size/shape (1–100 nm), linking closed pores to Na storage capacity.
X-ray Absorption Near Edge Structure (XANES)	Electronic Structure and Local Coordination	Probes electronic states, oxidation states, and local atomic environments.

**Table 2:** Exemplary strategies for carbon anode optimization and their performance advantages.

Research Direction	Key Parameters	Performance and Advantages
Closed-Pore Design	Pore size: <1 nm; Carbonization: 1100–2000 °C	Achieves 481.5 mAh/g capacity with 81% plateau contribution via optimized closed pores; 20% higher capacity than traditional hard carbon [77].
Pseudo-graphitic Domain Regulation	Interlayer spacing: 0.36–0.40 nm; Doping: 1–5 wt%;	Delivers 339 mAh/g total capacity (262 mAh/g from pseudo-graphitic domains) through expanded interlayer spacing, overcoming graphite limitations (<35 mAh/g) [69].
Defect Engineering	Pre-oxidation treatment; Low surface area	Enhances sloping capacity (>150 mAh/g) and ICE (>85%) via controlled defect generation; cost-effective for scalable production [70].
Sodiophilic Interface Modification	Ag loading: 1–10%	Reduces overpotential to 8 mV (vs. 23.6 mV for Cu and 12.5 mV for unmodified HC) and maintains stable cycling for 500 cycles with 493 mAh g^−1^ capacity retention; significantly outperforms unmodified HC (fails after 120 cycles) and Cu substrates (unstable deposition) [64].
Interlayer Spacing Control	Interlayer spacing: >0.4 nm; Solvent: Diglyme	Enables high-rate performance (~100 mAh/g@30 A/g) and 98% capacity retention over 8000 cycles; breaks graphite’s limitations [59].
Heteroatom Doping	Dopants (N/S/O/P); Carbonization: 1300–1600 °C	Boosts capacity by 15–20% and rate capability (>2C) through enhanced conductivity; Fe-catalyzed graphitization improves efficiency [90].

## Data Availability

No new data were created or analyzed in this study. Data sharing is not applicable to this article.
